# Dynamin-Related Protein 1 (Drp1) in Inflammatory Bowel Disease: Molecular Pathways Connecting Mitochondrial Dynamics with Intestinal Inflammation and Homeostasis

**DOI:** 10.3390/ijms27093828

**Published:** 2026-04-25

**Authors:** Yingying Chi, Hao Zhang, Chunbo Jia, Shujie Zeng, Xinyu Li, Dapeng Chen, Yong Ma

**Affiliations:** Department of Comparative Medicine, Dalian Medical University, Dalian 116044, China; bear_862042@163.com (Y.C.); shrh123467@163.com (H.Z.); jcb935359@163.com (C.J.); 13204091560@163.com (S.Z.); 15040832300@163.com (X.L.)

**Keywords:** inflammatory bowel disease, dynamin-related protein 1, mitochondrial fission, mitochondrial dynamics, intestinal epithelial barrier, inflammation

## Abstract

Inflammatory bowel disease (IBD) is characterized by chronic intestinal inflammation, epithelial barrier disruption and immune dysfunction. Alleviating and curing these pathological manifestations is the goal of IBD treatment. Despite substantial advances in targeted immunotherapies and anti-inflammatory strategies, achieving sustained intestinal mucosal healing remains a major clinical challenge. Dynamin-related protein 1 (Drp1) is a GTPase that mediates mitochondrial fission and plays a crucial role in maintaining the dynamic balance of mitochondrial morphology and function. In IBD, Drp1 expression is frequently upregulated and continuously activated, resulting in excessive fission and fragmentation of mitochondria. This mitochondrial dysregulation contributes to ATP depletion and excessive reactive oxygen species (ROS) production, thereby exacerbating disease progression and amplifying inflammatory signaling. This review highlights the distinctive role of Drp1 as an integrative node in IBD. Specifically, we connect mitochondrial dynamics with epithelial barrier failure, immune dysregulation, inflammatory cell death, and intestinal microenvironment remodeling. We further emphasize the potential relevance of Drp1 for biomarker-based patient stratification and mechanism-informed therapeutic targeting, thereby distinguishing this review from more descriptive accounts of mitochondrial dysfunction in intestinal inflammation.

## 1. Introduction

Inflammatory bowel disease (IBD) is a group of chronic, recurrent, and non-specific intestinal inflammatory disorders, mainly including ulcerative colitis (UC) and Crohn’s disease (CD). Epidemiological analyses indicate that between 1990 and 2017, the number of individuals affected by IBD increased from approximately 3.7 million to more than 6.8 million worldwide, and the total number of related deaths increased by 67%. Beyond its economic and healthcare burden on countries around the world, IBD significantly reduces patients’ quality of life, which has become an increasingly serious public health challenge [1]. With advances in research, IBD is increasingly recognized not as a disorder driven by a single immune abnormality, but as a systemic disease involving barrier dysfunction, immune–metabolic disturbance, and intestinal microbiota imbalance [2,3,4]. Accordingly, the identification of central molecular mediators capable of integrating metabolic and inflammatory signaling networks may provide critical insights into the complex pathophysiological basis of IBD.

Mitochondria serve as central platforms for integrating energy metabolism and inflammatory signaling, and they play a crucial role in maintaining intestinal homeostasis. The fission–fusion balance of mitochondria is essential for maintaining cell homeostasis, and its disruption frequently leads to increased inflammation, excessive production of reactive oxygen species (ROS), and reduced efficiency of ATP production. Upon activation by diverse upstream stimuli, dynamin-related protein 1 (Drp1) migrates from the cytosol to the mitochondrial outer membrane to initiate mitochondrial fission, thereby serving as a key regulator of mitochondrial dynamics [5]. Under physiological conditions, Drp1 contributes to mitochondrial quality control by maintaining the homeostatic balance of mitochondria. However, under inflammatory stress, the overactivation of Drp1 may act as an initiating event of mitochondrial dysfunction [6].

In recent years, accumulating evidence has shown that excessive mitochondrial fission and fragmentation occur in patients with IBD and in experimental mouse models, accompanied by a marked increase in the expression of Drp1. The mitochondrial dynamic imbalance mediated by overexpression of Drp1 is closely associated with a decline in mitochondrial membrane potential and the accumulation of mitochondrial reactive oxygen species (mtROS), and can further amplify inflammatory signaling through multiple pathways such as the NF-κB, MAPK and inflammasome pathways. In addition, Drp1 activation can also determine the fate of multiple cells such as PANoptosis, apoptosis, and ferroptosis, thereby modulating the functional states of intestinal epithelial cells(IECs), macrophages, and T lymphocytes, ultimately contributing to disorder and destruction of the intestinal microenvironment [7,8,9].

Therefore, Drp1 is not only a key mediator of mitochondrial fission but may also serve as an integrative hub coordinating metabolic stress responses and immune dysregulation in IBD. However, current discussions of Drp1 in intestinal inflammation remain largely fragmented across the mitochondrial biology, inflammatory signaling, and cell death pathways, and a more integrated framework linking these processes to epithelial barrier dysfunction, immune imbalance, and intestinal microenvironment remodeling is still lacking. In this review, we summarize the molecular structure, post-translational modifications, and regulatory mechanisms of Drp1, and further discuss its roles in intestinal inflammation, epithelial barrier injury, inflammatory cell death, and microenvironmental remodeling. Our aim is to provide a structured overview of how Drp1-centered mitochondrial dynamics may connect mechanistic pathology with biomarker stratification and therapeutic targeting in IBD.

## 2. Drp1: A Key Regulator of Mitochondrial Dynamics

### 2.1. Structure and Function of Drp1

Dynamin-related protein 1 (Drp1, gene name DNM1L) is a cytosolic GTPase with a molecular weight of approximately 80–100 kDa and belongs to the dynamin superfamily [10]. Drp1 was first discovered as an essential protein for mitochondrial fission in *Saccharomyces cerevisiae* and *Caenorhabditis elegans* [11], and then it has been confirmed that it also plays a very important role in mitochondrial fission of mammals [12,13]. Drp1 protein mainly exists in the cytoplasm and can also be transiently located in the outer membrane of mitochondria, where it plays an important role in mitochondrial fission and maintaining the dynamic balance of mitochondria.

**Figure 1:**
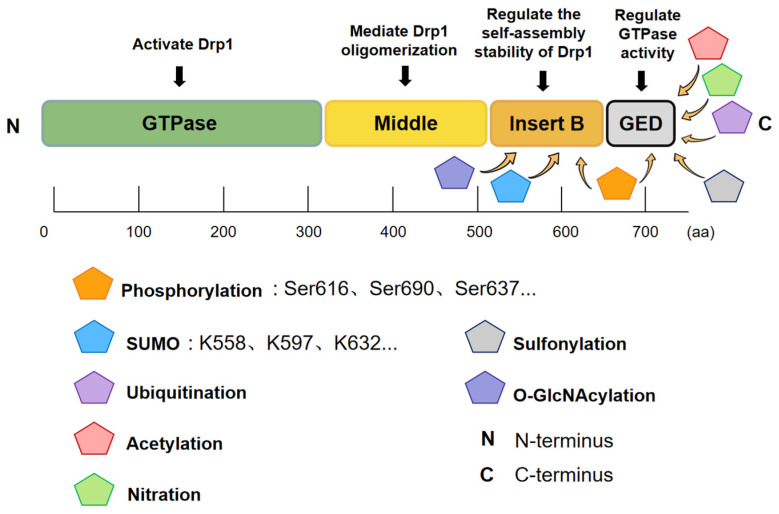
Molecular structure and post-translational modifications of Drp1.

Drp1 comprises four primary structural domains: an N-terminal GTPase domain (2–302aa), a middle domain (MD, 304–489aa), a variable Insert B region (B-insert, 502–640aa), and a C-terminal GTPase effector domain (GED, 644–735aa) [14,15,16]. Dimerization of the GTPase domain in the transition state of GTP hydrolysis is a critical step for Drp1 activation. The GTPase domain has a conserved structure in the dynamin superfamily—the classic Ras-like GTPase fold, namely an 8-strand β-sheet in the center surrounded by 7 α-helices and 2 short helices. GTPase mainly has five highly conserved nucleotide binding motifs (G1–G5), whose main function is to participate in the binding and hydrolysis of GTP, thereby driving conformational changes [17,18]. One of the characteristics that distinguishes Drp1 from other dynamins (such as Dynamin-1) is that the GTPase domain contains a unique 16-amino acid insertion sequence, forming a negatively charged flexible loop, but its specific function is not fully understood. It is speculated that it may be involved in conformational regulation or oligomerization. The middle domain mainly forms α-helical bundles, mediating the oligomerization of Drp1 and cooperating with the self-assembly stability of Drp1 with GED [14]. The GED participates in regulating GTPase activity and promoting polymerization, while the intermediate domain synergistically regulates GTPase activity, promoting oligomer stability and assembly ring formation [19]. Finally, the B-insert segment mainly regulates the binding of Drp1 to outer membrane receptors such as Mff and Mid49, and is related to various post-translational modifications [14]. We have drawn Figure 1 to visually illustrate the molecular structure of Drp1 and its common post-translational modification types and sites.

### 2.2. Structural Regulation and Functional Modulation of Drp1

Post-translational modifications of Drp1 play important roles in both its normal physiological functions and pathological states. Currently, researchers have employed various Drp1 mutant models to investigate how alterations in Drp1 affect different diseases. The following are several common modification types, supplemented with Table 1.

#### 2.2.1. Phosphorylation Is the Most Common Post-Translational Modification of Drp1

Ser616: Ser616 has been shown to be a Drp1 modification that can lead to excessive mitochondrial fission and damage [20]. Studies have shown that the Ser616 site, located near the GTPase domain, can be phosphorylated by ERK1/2 under mitosis or stress, thus enhancing the GTPase activity of Drp1, promoting its oligomerization and recruitment to the mitochondrial outer membrane, and ultimately triggering membrane constriction and fission. The authors knocked down Drp1 in cells to inhibit mitochondrial fission and then re-expressed two forms of Drp1 in this background: wild-type Drp1 (which can be phosphorylated) and the mutant Drp1S616A (which cannot be phosphorylated at the Ser616 site). It is found that only re-expression of wild-type Drp1 restored the excessive mitochondrial fission and fragmentation induced by oncogenic Ras, while the mutants expressing Drp1S616A could not restore mitochondrial division at all [21]. It is also shown in nerve cells that the activation of Cdk5 leads to excessive phosphorylation of Drp1 Ser616, which breaks the balance between mitochondrial fission and fusion, triggers excessive mitochondrial division, and eventually leads to the death of neurons [22].

Ser590: Ser-590 is also a Drp1 post-translational modification that promotes mitochondrial division. The phosphorylation of mouse Drp1 at the Ser-585 site (corresponding to human ser-590) promoted mitochondrial fission in mitotic cells. The authors found that the expression of non-phosphorylated mutant Drp1S585A in normal cells led to a significant reduction in mitochondrial fission in the mitotic period. In Drp1-knockdown cells, re-expressing wild-type Drp1 can restore mitochondrial fracture, but re-expression of the Drp1S585A mutant did not [23].

Ser637: Ser637 is a Drp1 modification that inhibits excessive mitochondrial fission. It suppresses the GTPase activity of Drp1 and prevents its recruitment to mitochondria, thereby reducing mitochondrial fission [24].

#### 2.2.2. SUMOylation and deSUMOylation

SUMOylation refers to the covalent attachment of small ubiquitin-like modifier proteins (SUMO1/2/3/4/5) to lysine residues of target proteins through a series of enzymatic reactions (E1–E2–E3), thereby altering the structure, localization, or activity of the target protein. DeSUMOylation is catalyzed by specific SUMO proteases (SENPs and SUMO-specific proteases) to restore the SUMO-modified protein to an unmodified state. According to a review by Wang et al. (2025), MAPL mediates SUMOylation of Drp1 at several sites within the VD region, including K532, thereby promoting the anchoring of Drp1 to mitochondria and facilitating mitochondrial fission [25,26,27]. SUMOylation of Drp1 can enhance mitochondrial fragmentation, converting the normal tubular mitochondrial network into numerous short rod-like fragments and markedly increasing the level of mitochondrial fragmentation. In addition, SUMOylation can inhibit proteasomal degradation of Drp1 and promote its recruitment to the mitochondrial outer membrane to execute the fission process [28]. Researchers have also found that once Drp1 reaches the mitochondria, SenP5 rapidly removes the SUMO modification (i.e., deSUMOylation), assembling Drp1 into active spiral oligomeric complexes. This rapid cycle of SUMOylation and deSUMOylation effectively promotes mitochondrial fission [29].

#### 2.2.3. Ubiquitination and Deubiquitination Modifications

Different enzymes can accelerate the degradation of Drp1 or play a stabilizing role through ubiquitination and deubiquitination respectively. For example, the E3 ubiquitin ligases Parkin and MITOL (also known as MARCH5/MARCHF5) can promote the degradation of Drp1 through ubiquitination [30,31], and the APC/C–Cdh1 complex suppresses mitochondrial fission during mitosis by ubiquitinating and degrading Drp1. Nevertheless, the deubiquitinating enzyme OTUD6A increases the stability of Drp1 in cells by removing ubiquitin residue on Drp1 [32].

**Table 1:** Other post-translational modifications of Drp1.

Type of Modification	Site	Function	References
Acetylation	Lysine 711 (K711) In GED	Promotes Drp1 oligomerization → ↑ mitochondrial fission → ↓ membrane potential, ↓ ATP, ↑ ROS → cell apoptosis	[33]
Deacetylation	-	SIRT3 binds Drp1 → removes K711 acetylation → inhibits excessive fission → improves mitochondrial function	[33]
Nitration	Two nitration sites, Tyr628 and Tyr665, are both located in the C-terminal GTPase effector domain (GED) and occur under inflammatory stress conditions.	Promotes Drp1 polymerization → ↑ mitochondrial recruitment and ring assembly → excessive fission → PINK1/Parkin-mediated mitophagy	[34]
O-GlcNAcylation	Threonine 585 (Thr-585) and threonine 586 (Thr-586) of Drp1	Inhibits Ser637 phosphorylation → ↑ GTP binding → Drp1 activation → mitochondrial fission	[35]
Sulfonylation	Cys-644	Activates Drp1 mtROS → endothelial senescence → vascular dysfunction	[36]

## 3. Mechanisms of Drp1 in the Pathogenesis of IBD

### 3.1. How Drp1 Contributes to the Pathogenesis of IBD

The current mainstream view is that the etiology of IBD remains complex and unclear, which is the result of the joint action of many factors such as genetic susceptibility factors, immune dysregulation, intestinal microbiota dysbiosis and impaired barrier function [37]. During the development of IBD, intestinal defense is weakened. Genetic susceptibility genes will lead to increased intestinal permeability and decreased barrier function, thereby allowing commensal bacteria to translocate across the mucosal barrier. For example, mutations in the intracellular pattern recognition receptor NOD2 reduce the secretion of Paneth cell antimicrobial peptides, impair its ability to clear bacteria, and inhibit the autophagy pathway, making intracellular bacteria more likely to survive. These intracellular bacteria will activate dendritic cells (DCs), promote their secretion of Th17-polarizing factors and inhibit the production of tolerogenic DCs, thereby breaking the balance of Treg/Th17 and causing adaptive immune dysregulation. Subsequently, the damaged epithelial cells release IL-25, IL-33 and other cytokines, which activate the innate lymphocytes to release IL-13 and IL-22 to further promote inflammation or repair. If the repair signal is insufficient and the inflammation signal (TNF-α) dominates, it will lead to mucosal ulcers and crypt abscesses in UC, or transmural inflammation in CD [38]. Some studies suggest that damage to the intestinal mucosal barrier should occur before the diagnosis of IBD [39,40], and there are experiments to support the repair of intestinal barrier damage as a potential drug target and worth exploring [41].

Mitochondria are not only the main organelles providing energy support to epithelial cells, but are also highly susceptible to stress, which can further amplify inflammation. Once the dynamic balance of mitochondria becomes dysregulated, the metabolism and barrier maintenance of epithelial cells will first become dysregulated, and then the immune response falls into a vicious cycle as the inflammatory response intensifies and cell death pressure increases. Studies have demonstrated that excessive mitochondrial fission may impair butyrate oxidation in colonic epithelial cells, increasing butyrate exposure in the colonic stem cell niche and further inhibiting mucosal repair in patients with UC [42]. Excessive mitochondrial fission is closely associated with a protein called Drp1, a critical GTPase mediating mitochondrial division. Classic cell biology evidence shows that Drp1 is primarily located in the cytosol but forms puncta on the mitochondrial surface that are associated with fission events. Purified Drp1 can also assemble into ring- or spiral-like oligomeric structures, which are consistent with its role in driving “constriction–division” mitochondrial fission [12]. In IECs of DSS-induced colitis and patients with UC, Drp1-mediated mitochondrial fission is enhanced and associated with disease severity, which can promote IEC ZBP1-dependent PANoptosis, directly linking mitochondrial stress events to the histopathological outcomes of epithelial shedding, ulceration, and inflammatory exacerbation. If the barrier damage occurs before diagnosis, the key to determining whether the barrier can be maintained or rapid re-epithelialization may not only be immune, but also the mitochondrial energy supply and the ability to cope with the stress of IEC. Drp1-driven excessive mitochondrial fission thus represents a critical mechanism by which metabolic dysfunction is translated into barrier instability [43].

### 3.2. Multiple Forms of Cell Death Induced by Excessive Drp1 Activation

#### 3.2.1. Apoptosis

In classical models of apoptosis, inhibition of Drp1 can block the transition of mitochondria from a tubular network to punctate fragments and significantly reduce key steps of the mitochondrial apoptosis pathway, such as membrane potential loss and cytochrome c release, thus reducing markers of apoptosis like TUNEL. This suggests that Drp1-mediated fission is an important amplification node of mitochondrial pathway apoptosis [44]. Mechanistically, excessive Drp1-mediated fission not only changes the morphology of mitochondria, but also reshapes the outer membrane of the mitochondria or changes the curvature to reduce the trigger threshold of mitochondrial outer membrane permeabilization (MOMP) under specific stress situations. In this way, the tBid-Bax axis is more prone to the oligopolymerization of Bax in the outer membrane, thus accelerating the leakage of cytochrome c and the activation of caspase-9/3 [45]. Meanwhile, the release of cytochrome c during apoptosis is also constrained by the cristae structure of the inner membrane. OPA1-mediated cristae remodeling determines whether cytochrome c sequestered within the cristae can be mobilized. Drp1-related fission and cristae remodeling are coupled in the stress state, so Drp1 overactivation is often accompanied by the loosening of OPA1-dependent cristae junctions, thus further exacerbating the mobilization and release of cytochrome c [46]. Overall, Drp1 accelerates the process of mitochondrial apoptosis by reducing the MOMP threshold of the outer membrane and influencing inner membrane cristae remodeling. In the context of IBD, this means that IEC is more sensitive to inflammatory stimuli such as TNF-α, hypoxia/oxidative stress, and is more prone to apoptosis, which leads to epithelial shedding and disruption of tight junctions, expanding the barrier gap and allowing more bacterial components to cross barriers, driving inflammation into a self-amplifying vicious cycle. However, under the persistent inflammatory stress of IBD, IEC death does not always occur through a single pathway. Drp1-mediated mitochondrial stress may also intersect with lipid peroxidation and inflammatory death platforms, promoting ferroptosis and PANoptosis.

#### 3.2.2. Ferroptosis

The core of ferroptosis lies in uncontrolled lipid peroxidation and the collapse of the GPX4/SLC7A11 axis, so that the lipid peroxidation chain reaction cannot be removed. In tumor models (OSCC), Zhen Wang et al. reported that inhibiting Drp1 promotes mitochondrial elongation and enhances glutaminolysis, thereby facilitating ferroptosis, indicating that Drp1 activity can influence cellular ferroptosis sensitivity by altering mitochondrial metabolic status [47]. In the context of IBD, He H (2025) [48] further applied this mechanism to IECs, showing that overactivation of the mechanosensitive channel Piezo1 causes Ca^2+^ overload and disrupts mitochondrial homeostasis, promoting epithelial ferroptosis. Electroacupuncture intervention maintained mitochondrial homeostasis and downregulated mitochondria-related molecular changes, thereby alleviating IEC ferroptosis and improving the IBD phenotype [48]. Existing evidence suggests that Drp1 or mitochondrial dynamics may alter IEC susceptibility to ferroptosis by affecting metabolic and oxidative stress buffering capacity, providing an additional cell death pathway contributing to barrier disruption.

#### 3.2.3. PANoptosis

Compared with single apoptosis or ferroptosis, IBD more commonly exhibits features of coupled inflammatory cell death pathways. Under the same inflammatory stress, the execution modules of apoptosis, necroptosis, and pyroptosis are simultaneously activated, turning cell death into a structural mechanism that drives barrier breakdown and sustains inflammation. In response to this phenomenon, recent reviews in the intestinal epithelium field have highlighted that IECs in IBD show crossactivation and backup between multiple death pathways. PANoptosis, as an integrated cell death framework, better explains the persistence and amplification of mucosal injury and suggests potential therapeutic value [49].

Ye Z et al. reported in UC patient samples and DSS-induced colitis models that IECs exhibit a “mitochondrial fission (Drp1)—mtROS—ZBP1 dependent PANoptosis” axis, which positively correlates with disease severity. Genetic or pharmacological interventions that reduce Drp1-mediated fission signals attenuate IEC death and colitis phenotypes. Saquinavir was also shown to inhibit this process via a Drp1-related mechanism, indicating that excessive Drp1-mediated fission serves as a critical hub converting mitochondrial stress into “barrier disruption + inflammatory amplification” [8]. Furthermore, the role of ZBP1 as an innate sensor and assembly node in the intestinal epithelium has been independently supported. A study published in *Cell* in 2024 reported that Z-nucleic acids formed after tissue injury are recognized by ZBP1, which then drives epithelial inflammatory cell death via caspase-8 and delays repair in the colon and small intestine. This type III IFN–ZBP1–caspase-8 axis is also active in patients with IBD, suggesting that the “interferon tension–ZBP1–inflammatory death” module is a repeatedly invoked damage pathway in the intestinal mucosal inflammatory environment [50].

Thus, excessive mitochondrial fission resulting from Drp1 overexpression or hyperactivation is far from a bystander in IBD progression. On one hand, it weakens the energy supply and repair capacity of IECs; on the other hand, it drives epithelial shedding and ulcer expansion through mtROS production and inflammatory death pathways such as the ZBP1–PANoptosis axis, ultimately promoting disease progression within a self-perpetuating loop of “barrier breach—microbial translocation—inflammatory amplification.”

**Figure 2:**
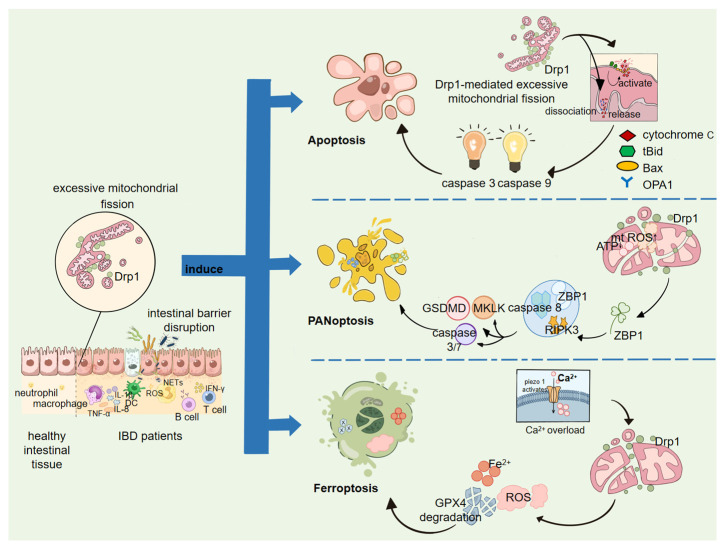
Signaling pathways by which Drp1 activation-mediated excessive mitochondrial fission induces cell death in IBD, including apoptosis, PANoptosis, and ferroptosis. ATP ↓ and ROS ↑ indicate that after Drp1 is recruited to the mitochondria, excessive mitochondrial fission begins, leading to decreased ATP production efficiency and increased ROS generation.

We have prepared Figure 2 to provide a visual representation of the three distinct cell death modalities.

### 3.3. Cascade Amplification Mechanisms of Drp1-Induced Intestinal Barrier Damage

Drp1 is a key regulator of mitochondrial fission. In IECs from patients with UC and DSS-induced colitis models, Drp1 expression is upregulated and drives excessive mitochondrial fission and fragmentation by binding to mitochondrial membrane receptor proteins such as Fis1 and Mff. Transmission electron microscopy reveals shortened mitochondria with increased density, accompanied by reduced ATP production and loss of mitochondrial membrane potential (ΔΨm). This alteration is considered an upstream event in IBD because mitochondrial homeostasis in epithelial cells plays a key role in barrier maintenance and inflammatory threshold control. Once fission becomes excessive and the mitochondrial network fragments, IECs and mitochondria rapidly enter a mutually reinforcing cascade of damage [51].

Specifically, reduced mitochondrial connectivity imposes an immediate bioenergetic cost, including impaired respiratory efficiency, loss of ΔΨm, and decreased ATP supply, thereby weakening epithelial repair, tight-junction integrity, and ion transport and rendering the barrier more susceptible to breach. Fragmented mitochondria also exhibit inefficient electron transport and enhanced electron leakage, resulting in rapid mtROS accumulation. These mtROS not only damage mitochondrial components, but also act as amplification signals that intensify mucosal inflammatory stress. In parallel, oxidative stress and structural fragility promote the release of mtDNA and associated mitochondrial damage-associated molecular patterns (mtDAMPs), thereby extending local mitochondrial injury into sustained immune amplification [52]. Within this cascade, mtDAMPs mainly contribute to immune amplification, whereas PANoptosis acts as a direct execution layer of barrier collapse.

Within this cascade, mtROS act as a decisive amplifier, pushing mitochondrial fragmentation toward a more complex, mixed-mode cell death [53]. Ye Z et al. demonstrated that IEC-specific Drp1 heterozygous knockout mice (Drp1-hetCKO) exhibited significantly lower mtROS levels compared with wild-type mice, which correlated negatively with PANoptosis marker expression [8]. Mechanistically, mtROS can oxidize the Cys327 residue of ZBP1 (Z-DNA binding protein 1) to form a sulfenic acid (-SOH) modification, inducing conformational changes and promoting ZBP1 oligomerization to assemble the PANoptosome. This process is independent of ZBP1’s Zα domain (Z-nucleic acid recognition) but requires the RHIM domain for interaction with RIPK3 [54]. Subsequently, ZBP1 recruits RIPK3, caspase-8, and other effectors via its RHIM domain, forming a PANoptosome that simultaneously engages the apoptosis (caspase-3/7), pyroptosis (GSDMD/GSDME), and necroptosis (MLKL) pathways, ultimately triggering PANoptosis [55]. Extensive lytic IEC death leads to loss of barrier integrity, release of DAMPs and pro-inflammatory cytokines, and recruitment of inflammatory cells, thereby establishing a self-reinforcing “fission–ROS–death–inflammation” loop that sustains mucosal injury in IBD.

## 4. Relationship Between Drp1-Mediated Mitochondrial Fission and Inflammatory Responses in IBD

### 4.1. Drp1-Mediated Mitochondrial Fission Represents a Common Mechanism Underlying Inflammatory Responses in IBD

The intestinal microenvironment in IBD is characterized by dysbiosis, altered metabolites, immune dysregulation, and intestinal barrier damage. These alterations also represent key targets for comprehensive therapeutic intervention.

In terms of innate immunity, under the influence of various intestinal microorganisms and metabolite signals in IBD, the M1/M2 polarization balance of macrophages is broken, and M1 macrophages are overactivated and secrete high levels of pro-inflammatory factors, which exacerbate local inflammatory reactions. At the same time, neutrophils recruited by M1 macrophages will excessively release ROS and NETs, aggravating intestinal tissue damage. The activation of DCs is based on macrophages and neutrophils, presenting abnormal antigen presentation and promoting Th1/Th17 differentiation.

In adaptive immunity, the excessive activation of Th1 and Th17 cells leads to the secretion of large amounts of pro-inflammatory cytokines such as IFN-γ and IL-1. Concurrently, reduced abundance and impaired function of Treg cells result in uncontrolled inflammation. Abnormal B cell and plasma cell activity, including increased IgG secretion and complement system activation, further aggravate immune dysregulation in the gut. The disruption of immune cell abundance and function, together with the accumulation of inflammatory mediators, constitutes the intestinal immune microenvironment in IBD. This immune imbalance, combined with dysbiosis and barrier damage, forms a positive feedback loop that drives the persistent progression of inflammation [56].

Multiple studies have now demonstrated that Drp1 expression is markedly upregulated during IBD progression, with Drp1-mediated excessive mitochondrial fission serving as a central link between inflammatory stimuli and cellular dysfunction [43]. Multiple studies support a link between Drp1 upregulation and inflammation-associated mitochondrial dysfunction in IBD. These observations suggest that Drp1-mediated fission may connect inflammatory stress with epithelial injury and immune amplification, although the mechanistic strength of evidence differs across cellular contexts [7,43,57].

Below, we detail the relationship between Drp1 and IBD-associated inflammation, focusing on Drp1-induced changes in various immune cell populations, pro-inflammatory cytokine production, and the associated signaling pathways. Notably, the current evidence is not equally strong across these mechanisms. The most direct support comes from IECS, where Drp1-mediated mitochondrial fission has been linked to barrier disruption and inflammatory cell death in both experimental colitis models and patient samples. By comparison, the available evidence in macrophages is more consistent with a role in inflammatory amplification, whereas the evidence in T cells remains more indirect. In addition, we have prepared Table 2 to provide a summary.

### 4.2. Drp1 Regulates Intestinal Innate Immunity and Contributes to Chronic Intestinal Inflammation

#### 4.2.1. Drp1-Mediated Mitochondrial Fission Promotes Inflammatory Responses in Intestinal Epithelial Cells and Disrupts the Epithelial Barrier

IECs are essential for maintaining gut barrier integrity and preserving the homeostasis of the intestinal microbiota, metabolites, and immune microenvironment. Barrier disruption and inflammatory responses arising from IEC injury are important pathological roots and characteristics of the progression of IBD. Recent studies in both animal models and humans have shown that Drp1-mediated mitochondrial fission in IECs is closely linked to cellular damage and inflammatory signaling. We emphasize that Drp1 not only regulates mitochondrial morphology in IECs, but also acts as a central hub linking inflammatory signaling, metabolic disturbance, and cell death pathways.

Inflammatory stimuli promote sustained mitochondrial fission in IECs by modulating the phosphorylation state of Drp1, leading to mitochondrial fragmentation and dysfunction. Under LPS stimulation, Drp1 is activated by dephosphorylation at the Ser637 site, and is translocated from the cytosol to mitochondria, which eventually leads to impaired mitochondrial function, decreased respiratory capacity (OCR), reduced membrane potential (ΔΨm), and mitochondrial fragmentation. It is worth noting that damaged mitochondria produce excessive mtROS, activating classical pro-inflammatory signaling pathways such as NF-κB and MAPK, which directly leads to a large number of pro-inflammatory factors (TNF-α, IL-1β, and IL-6) and inflammatory enzymes (iNOS and COX-2). Consequently, the barrier function of the injured IECs is compromised, manifesting as a “leaky gut” phenotype. Other studies have also shown that phosphorylation of Drp1 at Ser616 in IECs activates Drp1, drives excessive mitochondrial fission, inhibits butyrate metabolism, and thereby suppresses mucosal repair [7].

Besides classical inflammatory signaling pathways, Drp1-mediated mitochondrial fission can amplify IBD inflammation by inducing PANoptosis in IECs. Zhiming Ye et al. used DSS-induced colitis mouse models and IEC-specific Drp1 heterozygous knockout mice (Drp1-hetCKO) and found that excessive mitochondrial fission in IECs generated mtROS, which oxidatively modified the innate immune sensor ZBP1 at Cys327, triggering ZBP1-dependent PANoptosis. This led to epithelial barrier disruption and worsened colitis. The anti-HIV drug saquinavir (SQV) could directly bind and inhibit Drp1, blocking this process and alleviating colitis. Clinical sample analysis further confirmed that Drp1 expression in IECs positively correlated with disease severity, as well as with NLRP3, pMLKL, Mayo scores, and especially ZBP1, indicating that Drp1 is associated with PANoptosis and contributes to IBD pathogenesis [8].

Additionally, ferroptosis in IECs during IBD may also be linked to Drp1 activation. In the IBD pathological microenvironment, abnormal mechanical stress overactivates the Piezo1 channel, causing calcium influx, mitochondrial calcium overload, and oxidative stress, which significantly upregulates Drp1, leading to excessive mitochondrial fission, structural damage, and functional loss. This mitochondrial homeostasis imbalance further weakens the antioxidant defense of cells (such as GPX4 decline and GSH depletion), and promotes lipid peroxidation and iron accumulation, ultimately triggering ferroptosis and damaging the intestinal epithelial barrier [48].

In summary, there is currently much evidence that Drp1-mediated mitochondrial fission abnormalities can lead to damage to IECs and cascade amplification of inflammatory reactions through the PANoptosis or ferroptosis pathway. Interventions such as electroacupuncture, which inhibits Piezo1 activity and downregulates Drp1 expression; the P110 peptide, which inhibits Drp1 activation; or the drug saquinavir (SQV), which directly binds and inhibits Drp1, can reverse these processes and restore epithelial homeostasis. Taken together, the currently available data in IECs provide a particularly direct mechanistic link between Drp1-mediated mitochondrial fission, barrier dysfunction, and inflammatory injury in IBD. These findings also support intestinal Drp1 inhibition as a potential therapeutic strategy.

#### 4.2.2. Drp1 Maintains the Pro-Inflammatory Phenotype of Macrophages and Amplifies the Inflammatory Response

Multiple human and animal experiments have demonstrated that macrophages play an important role in the development of chronic colonic inflammation in UC and CD, showing a typical M1 inflammatory phenotype. Differentiated macrophages activate signaling pathways such as IL-8, IFN-γ, TNF-α, and TREM1, thus aggravating intestinal inflammation. Simultaneously, they promote the activation of inflammasomes such as NLRP3, and facilitate the maturation and release of cytokines including IL-1β and caspase-1 (CASP1). These macrophages also secrete chemokines that recruit neutrophils, T cells, and other immune cells to the sites of intestinal lesions [9,58].

Recent studies have shown that Drp1 is activated in the inflammatory environment induced by LPS, promoting mitochondrial fission in macrophages and facilitating a metabolic shift towards glycolysis, which enhances the production of pro-inflammatory cytokines such as TNF-α, IL-6, and IL-1β. Using Drp1 inhibitors such as Mdivi-1 or Drp1 gene silencing has been shown to reduce inflammatory responses [59]. Further investigations have revealed that Drp1 can regulate mitochondrial immune signaling pathways by modulating mitochondrial morphology and function in macrophages. For example, Drp1 specifically promotes the post-transcriptional production of TNF-α through the regulation of mitochondrial fission, thereby enhancing macrophage secretion of TNF-α. Drp1-mediated mitochondrial fission may also affect macrophage stress responses, indirectly regulating the translational mechanisms of TNF-α. Using shRNA to knock down Drp1 (Drp1KD) can reduce TNF-α release by macrophages under LPS stimulation, suggesting that Drp1 could serve as a potential therapeutic target for inflammatory diseases [60].

Notably, in DSS-induced colitis models, the level of mtROS in macrophages is significantly increased, promoting the binding between TXNIP and NLRP3, which activates caspase-1 and subsequently cleaves the precursors of IL-1β and IL-18, facilitating their maturation and release. Using mitochondria-targeted antioxidant MitoQ can inhibit NLRP3 inflammasome activation by removing mtROS, thus reducing IL-1β and IL-18 expression and alleviating colitis [61]. According to this, we hypothesize the existence of a Drp1–mtROS–inflammasome axis that synergistically amplifies the pro-inflammatory response of macrophages.

Drp1 may maintain the pro-inflammatory phenotype of macrophages by directly modulating cytokine secretion and through a second mechanism involving mtROS generation and inflammasome activation. Interventions targeting Drp1, such as Mdivi-1, or combined strategies aimed at mtROS clearance, like MitoQ, may provide new ideas for multi-targeted therapy in IBD. However, the specific mechanisms and therapeutic potential of Drp1 in IBD require further investigation. Compared with the evidence in IECs, the current support for Drp1-mediated mechanisms in macrophages is more focused on inflammatory amplification than on direct execution of barrier injury.

### 4.3. Drp1 Modulates Adaptive Immunity and Disrupts Intestinal Immune Tolerance

#### 4.3.1. Drp1 Participates in Metabolic Reprogramming During T Cell Activation to Sustain T Cell Function and Effector

T cells are the core adaptive immune effector cells of intestinal immune tolerance and inflammatory responses, and their activation status and function are particularly critical in IBD. Accumulating evidence indicates that both the differentiation spectrum and functional effects of T cells are markedly altered in IBD. In CD, Th1 and Th17 cells are increased significantly, secreting a large amount of IFN-γ and IL-17, whereas UC is primarily mediated by Th2-like responses, characterized by the production of pro-inflammatory cytokines such as IL-4, IL-5, and IL-13, accompanied by enhanced Th17 cell response [62,63,64]. Moreover, the imbalance of Treg/Th17 cells constitutes a key factor exacerbating intestinal inflammation in IBD. If activated CD4+T cells are transplanted into immunodeficient mice, severe colitis will occur in the receptor mice, which verifies the driving role of Th cells in the development of IBD [62,65].

In recent years, mitochondrial dynamics, especially Drp1-mediated mitochondrial fission, have been considered a critical hub connecting T cell metabolic states with their phenotype, which may influence T cell-mediated IBD pathogenesis. In IBD, activated T cells will undergo metabolic reprogramming and turn to aerobic glycolysis (the Warburg effect), preferentially utilizing glycolysis even under normoxic conditions to rapidly generate ATP and biosynthetic precursors. Drp1 plays an important role in maintaining the normal processes of mitochondrial fission and promoting glycolytic metabolism in T cells, and supporting T cells to execute functions [66,67].

Although direct evidence demonstrating that Drp1 specifically regulates T cell function in IBD is currently lacking, studies from various autoimmune disease models provide important clues regarding its potential role. In rheumatoid arthritis (RA), an autoimmune disease analogous to IBD, it has been shown that PD-1 signaling inhibits hypoxia-inducible factor HIF-1α and downregulates the expression of the mitochondrial fission protein Drp1. Ultimately, mitophagy is impaired, leading to the accumulation of dysfunctional mitochondria and excessive mtROS production. This process facilitates senescence-associated secretory phenotype (SASP) and the release of cytotoxic molecules, exacerbating joint inflammation and tissue destruction. This shows the critical importance of normal Drp1 expression in maintaining T cell homeostasis [68]. In chronically aged or functionally impaired T cells, proper Drp1 expression is indispensable for sustaining normal mitochondrial autophagy and cellular homeostasis.

Conversely, in disease models characterized by overactivation of T cells, such as multiple sclerosis, inhibiting Drp1 phosphorylation with Mdivi-1 can suppress mitochondrial fission, and then reduce ROS production and attenuate excessive T cell activation to regulate immune responses [69]. It shows that in acutely activated T cells, overactive Drp1 drives excessive mitochondrial fission and ROS production, which eventually leads to exacerbated inflammation. In chronic inflammatory or aged environments, Drp1 is essential for maintaining the normal mitochondrial fission and mitophagy.

Based on the existing evidence, we can infer that Drp1 promotes T cell glycolysis. This is crucial for maintaining the activation of effector T cells, Th17 cells, and Th1 cells, which often play important roles in the development of IBD. Although direct research targeting IBD remains limited, the role of Drp1 has been studied increasingly in many autoimmune diseases that are highly similar to IBD, providing important clues for Drp1 to explain the adaptive immune dysregulation in IBD from the perspective of metabolism and mitochondrial homeostasis. Nevertheless, compared with the evidence in IECs and macrophages, the current support for Drp1-mediated T cell dysregulation in IBD remains more indirect and relies partly on extrapolation from other autoimmune disease models.

#### 4.3.2. Drp1 in B Cells: Potential for Future Research

B cells have long been considered to play a secondary role in the pathogenesis of IBD. However, increasing evidence indicates that distinct B cell subsets are involved in maintaining intestinal immune homeostasis. Research has shown that mice deficient in B cells develop more severe colitis, highlighting the protective role of B cells in intestinal inflammation. In particular, regulatory B cells producing IL-10 and plasma cells secreting IgA are critical for suppressing excessive immune responses in the gut [70,71]. Although the role of Drp1-mediated mitochondrial dynamics in B cells in IBD has not yet been directly investigated, mitochondrial metabolism is increasingly recognized as a key regulator of B cell activation and differentiation, suggesting a potential but as yet unexplored link [72].

**Table 2:** Cell type-specific roles of Drp1-mediated mitochondrial fission in IBD-associated inflammation.

Cell Type	Drp1 Activation Status	Key Mechanisms	Key Molecules	Contribution to IBD Inflammation	References
Intestinal epithelial cells (IECs)	Expression ↑; phosphorylation at Ser616 ↑; dephosphorylation at Ser637 ↑	Excessive mitochondrial fission → ATP ↓, mtROS ↑ → barrier disruption (tight junction loss, “leaky gut”) → PANoptosis (ZBP1-dependent) and ferroptosis	ZBP1-PANoptosome, NF-κB, MAPK, Piezo1-Ca^2+^, GPX4	Epithelial shedding, ulceration, mucosal repair failure, amplification of inflammation	[7,8,48]
Macrophages	LPS-induced activation; mitochondrial fission enhanced	Metabolic reprogramming (glycolysis ↑) → pro-inflammatory cytokine secretion (TNF-α, IL-6, IL-1β) → NLRP3 inflammasome activation	TLR4-MyD88, PGAM5-Drp1, TXNIP-NLRP3, mtROS	Maintenance of M1 pro-inflammatory phenotype, amplification of intestinal inflammation	[9,58,59,60,61]
T cells	Regulates mitochondrial fission and mitophagy	Supports T cell activation, proliferation, and effector functions (Th1/Th17); metabolic reprogramming (glycolysis)	HIF-1α, cMyc, PD-1 signaling	Inferred from RA/MS models; promotes adaptive immune dysregulation in IBD (requires direct validation)	[62,63,64,65,66,67,68,69]
B cells	Limited evidence; likely present	Potentially influences B cell differentiation, IgA secretion, and IL-10 production	Under investigation	Currently unknown; represents a future research direction	[70,71,72]

## 5. Drp1 Influences the Intestinal Microenvironment

### 5.1. Gut Microbiota Dysbiosis Exacerbates the Overactivation of Drp1

The intestinal microbiota represents an important upstream source of inflammatory stimulation; more importantly, microbial metabolites can continuously regulate the modes of energy supply and mitochondrial homeostasis in IECs and mucosal immune cells at an upstream regulatory level, thereby shaping intestinal barrier function and the inflammatory threshold [73]. Dysbiosis does not merely alter microbial composition or metabolite availability, but can also reshape mucosal immune homeostasis and epithelial stress responses in IBD. This provides an upstream mechanistic context in which Drp1-associated mitochondrial remodeling may shift from adaptive regulation toward maladaptive inflammatory amplification [74]. Among the diverse classes of microbial metabolites, short-chain fatty acids (SCFAs), particularly butyrate, can act as key chemical signals linking “microbial community structure–host metabolism–intestinal barrier function.” Butyrate not only serves as a major energy substrate for colonic epithelial cells and influences the metabolic foundation of these cells, but can also modify immune response thresholds and mitochondrial quality-control networks through receptor-mediated signaling and epigenetic regulation, thereby converting alterations in microbial metabolism into metabolic changes in host cells [73,75]. Butyrate can reshape cellular energy status, including parameters such as the ATP/AMP ratio and substrate utilization spectrum, thereby driving the activation of the energy-sensing hub AMP-activated protein kinase (AMPK), which functions as an important molecular switch that translates cellular energy states into mitochondrial dynamic remodeling [76]. AMPK can enhance the recruitment of Drp1 by phosphorylating the mitochondrial outer membrane receptor mitochondrial fission factor (MFF), thereby promoting mitochondrial fission through the AMPK → MFF → Drp1 axis and enabling energy-sensing signals to directly converge on the remodeling of mitochondrial morphology and function [76,77]. In this process, MFF acts as the mitochondrial outer membrane recruitment receptor for Drp1, and AMPK-mediated phosphorylation of MFF enhances the localization and accumulation of Drp1 on the mitochondrial outer membrane, ultimately leading to observable mitochondrial fission events [76].

It should be noted that the influence of microbial metabolites on Drp1 is generally not unidirectional and may exert context-dependent regulatory effects. Under different conditions, including variations in metabolite concentration, cellular background, and redox microenvironment, butyrate may regulate mitochondrial-related genes and mitochondrial dynamic regulators (including Drp1 and FIS1) in different directions, suggesting that this process is highly dependent on the microenvironment rather than functioning as a fixed and unidirectional molecular switch [78]. In pathological contexts that more closely resemble intestinal disease, studies have further observed that butyrate can alleviate epithelial mitochondrial morphological disruption induced by pro-inflammatory bacteria, such as adherent-invasive Escherichia coli (AIEC), and improve intestinal barrier function, thereby providing additional support for the conceptual framework linking “butyrate–mitochondrial morphology–intestinal barrier function” [79].

Under conditions of gut microbiota dysbiosis, excessive activation of Drp1 may be further promoted through at least two mutually interacting amplification pathways. The first pathway arises from an imbalance in the mitochondrial dynamics network under a low-butyrate environment: reductions in butyrate-producing bacteria and alterations in the SCFA profile can shift IECs from a butyrate-supported oxidative metabolic mode toward a state of energy stress. When this transition occurs concurrently with inflammatory or stress conditions, the AMPK–MFF/Drp1-mediated remodeling of mitochondrial dynamics may shift from adaptive regulation toward excessive mitochondrial fission and fragmentation of the mitochondrial network, subsequently resulting in reduced ATP production, impaired repair capacity, and increased vulnerability of the epithelial barrier, thereby providing a metabolic basis for the persistence of inflammation [75,76]. Another proposed pathway suggests that excessive mitochondrial fission can reduce the efficiency of the electron transport chain and increase mtROS generation. On the one hand, mtROS can damage tight junction structures and epithelial integrity; on the other hand, mtROS may function as an inflammatory amplification signal that promotes mucosal immune activation, ultimately altering the intestinal luminal environment and, in turn, further disturbing microbial community structure, thereby forming a potential positive feedback loop [80]. Therefore, studies and reviews in the field of IBD increasingly regard mitochondrial dysfunction and disturbances in mitochondrial dynamics as important pathological foundations of intestinal inflammation and potential therapeutic targets [43,75]. Recent studies have more directly suggested that Drp1-mediated mitochondrial fission in IECs can exacerbate inflammatory responses and may possess therapeutic target potential [7]. At the molecular readout level, the activation state of Drp1-mediated fission is often characterized by site-specific phosphorylation: phosphorylation at Ser616 is generally associated with a pro-fission state, whereas phosphorylation at Ser637 is commonly considered to be related to inhibitory regulation. These changes are frequently accompanied by enhanced recruitment of Drp1 to the mitochondrial outer membrane and fragmentation of the mitochondrial network [81].

### 5.2. Drp1 and the Intestinal Microenvironment: mtROS, Metabolic Reprogramming, and Microecological Feedback

Based on the above “microbial metabolic input–AMPK–MFF/Drp1–mitochondrial fission” axis, the reason why Drp1-mediated abnormalities in mitochondrial dynamics can be amplified at the tissue level into intestinal microenvironmental imbalance lies in the fact that this process simultaneously reshapes redox signaling (mtROS) and metabolic flux allocation (OXPHOS and glycolysis), and further remodels the intestinal luminal ecological niche through alterations in epithelial barrier function and immune activation thresholds, thereby forming a triangular closed loop of “microbiota–metabolism–immunity.”

#### 5.2.1. How Excessive Drp1-Mediated Mitochondrial Fission Drives the mtROS–Inflammation Amplification Axis

Excessive Drp1-mediated mitochondrial fission can lead to fragmentation of the mitochondrial network, reduced electron transport efficiency, and instability of mitochondrial membrane potential, thereby promoting mtROS accumulation. In the intestinal mucosa, these mtROS function not only as indicators of mitochondrial stress but also as amplification signals within the local microenvironment. Studies have shown that Drp1 activation is accompanied by increased mtROS levels, impaired barrier function, and alterations in microbial composition, supporting the possibility that a “Drp1–mtROS–barrier–microecology” feedback loop can occur in vivo [80]. In this context, the significance of Drp1 lies not merely in its ability to promote epithelial injury, but also in its potential to convert mitochondrial stress into tissue-level ecological instability. Once barrier integrity declines and mucosal oxygen handling is altered, the luminal niche may shift in ways that favor microbiota imbalance, thereby feeding back to sustain inflammation and epithelial stress.

It is noteworthy that excessive activation of Drp1 is often accompanied by redistribution of metabolic flux (for example, enhanced glycolysis), and this coupling between “Drp1-driven mitochondrial dynamics and metabolic reprogramming” has also been directly supported in other epithelial stress models. For instance, in hyperoxia-induced alveolar type II epithelial cells (ATII), Drp1 activation promotes mitochondrial fission and drives glycolytic reprogramming.

#### 5.2.2. How Drp1 Drives Metabolic Reprogramming

Excessive mitochondrial fission mediated by Drp1 does not merely result in morphological fragmentation. More importantly, it alters the structural and functional coupling of the mitochondrial respiratory chain. When the mitochondrial network shifts from a fusion-dominant state toward a fission-dominant state, the efficiency of electron transport and the stability of the mitochondrial membrane potential become more prone to fluctuation. This shift drives the oxidative phosphorylation (OXPHOS)-based energy production mode toward a stress-associated metabolic program, allowing transient immune stimuli to be more readily consolidated into a sustained pro-inflammatory metabolic background. In innate immune cells, this pathway has a relatively well-defined upstream signaling entry point: LPS can rapidly induce Drp1-associated mitochondrial dynamic remodeling through the TLR4—MyD88 signaling axis, accompanied by enhanced inflammatory responses. At the same time, regulation of Drp1 under LPS stimulation exhibits site-specific dynamics (initial phosphorylation followed by dephosphorylation), suggesting that Drp1 resides at a proximal node linking “inflammatory signaling—mitochondrial events” [82]. Based on this proximal node, metabolic-level evidence further indicates that Drp1 participates in shaping the structural state of the respiratory chain. During LPS-induced immunometabolic reprogramming, deletion or knockdown of Drp1 alters the relative abundance of electron transport chain (ETC) components and ATP synthase-associated subunits and remodels the OXPHOS protein profile, thereby extending the effects of fission-dominant mitochondrial network fragmentation to the level of respiratory chain assembly and bioenergetic programming. Specifically, under LPS-induced immunometabolic reprogramming, Drp1 knockdown not only changes mitochondrial morphology but is also accompanied by rearrangement in the relative abundance and composition of oxidative phosphorylation complexes (I–V) and ATP synthase-related subunits, with corresponding remodeling of the respiratory chain-associated protein spectrum and respiratory output, suggesting that Drp1 may regulate energy production modes by influencing the structural organization of the respiratory chain and thereby affect the direction of metabolic programming [60].

At the molecular mechanistic level, the PGAM5–Drp1 axis links mitochondrial fission, mtROS, and inflammatory signaling: PGAM5 can form a complex with Drp1 and regulate its dephosphorylation, thereby promoting mtROS generation and engaging pathways such as NF-κB and MAPK, leading to pro-inflammatory cytokine expression, such that the sequence “fission → mtROS → inflammation amplification → metabolic bias” manifests as a nodal and potentially targetable mechanistic structure [83]. Furthermore, under specific stress conditions, the coupling between enhanced mitochondrial fission and elevated glycolysis has been associated with the phosphorylation/dephosphorylation status of Drp1 (Ser616/Ser637), suggesting that site-specific Drp1 states may be linked to both glycolytic enhancement and fission phenotypes [84]. Therefore, when persistent inflammation and energy stress coexist, IECs are more likely to shift from an OXPHOS-dominant homeostatic energy supply toward compensatory glycolysis and energy-vulnerable states, thereby reducing the energetic reserve required for repair and barrier maintenance and providing a metabolic foundation for a pro-inflammatory microenvironment [75].

Finally, it should be noted that when using pharmacological evidence to support the causal role of Drp1 in metabolic reprogramming, over-extrapolation should be avoided. The commonly used Drp1 inhibitor, Mdivi-1, under many conditions primarily acts as a reversible inhibitor of mitochondrial complex I rather than a specific inhibitor of Drp1 GTPase. Therefore, when employing Mdivi-1 as key evidence, it should be corroborated wherever possible with genetic interventions and validations of Drp1 localization and activation status [85].

### 5.3. Ecological Niche Feedback: The “Microbiota–Metabolism–Immunity” Triangle

The closure of the butyrate input axis with the mtROS/metabolism axis relies on the recognition that the metabolic state of intestinal epithelial mitochondria inherently shapes the luminal ecological niche (oxygen gradient, substrate availability, and mucus-layer microenvironment). Therefore, mitochondrial dynamic events not only respond to microbial metabolites but may also reciprocally modify the survival conditions of the microbiota. Specifically, butyrate, as a major energy substrate for colonic epithelial cells, can be oxidatively metabolized by these cells to enhance mucosal oxygen consumption and maintain a “physiological hypoxia” gradient, thereby restricting competitive expansion of facultative anaerobes and stabilizing a community structure dominated by obligate anaerobes. The “butyrate–epithelial OXPHOS/oxygen consumption–hypoxic niche” thus constitutes an important physicochemical foundation for microbial homeostasis [86,87].

Accordingly, under inflammatory or stress conditions, excessive Drp1-mediated fission accompanied by mtROS accumulation, impaired OXPHOS output, or metabolic program shifts often coincides with reduced epithelial energy reserve and reparative capacity, barrier vulnerability, and altered inflammatory mediator profiles. These changes are not merely manifestations of tissue damage but may also, by decreasing mucosal oxygen consumption and increasing luminal availability of electron acceptors/oxygen, drive the luminal environment from strict anaerobiosis toward relative oxygenation, thereby providing an ecological advantage for facultative anaerobes and promoting shifts in microbiota composition and metabolic profiles (for example, decreased SCFA production and increased respiratory advantage using nitrate/oxygen as electron acceptors). This “oxygen availability-driven imbalance” has been systematically proposed and repeatedly validated across multiple inflammatory models, representing a key bridge linking “host metabolic events” to “microbiota structural changes” [88,89]. Within this closed loop, the role of Drp1 is to concretize mitochondrial changes into an amplifiable driving node: when Drp1-mediated fission is enhanced, leading to mtROS accumulation and a decline in barrier readouts, the luminal ecological niche is concomitantly altered and may be accompanied by microbiota composition shifts, thus providing in vivo support for a “dynamics–ROS/metabolism–barrier–microecology” feedback structure [80]. Furthermore, recent in situ intestinal studies have emphasized that mitochondrial alterations can be both outcomes and drivers: epithelial mitochondrial perturbations alone can induce microbiota-dependent tissue damage and inflammation-associated gene expression, indicating a strong potential coupling between host mitochondrial status and the microbiota [90].

Therefore, the microbiota–metabolism–immunity system can be conceptualized as a triangular model: microbial metabolites (such as butyrate) influence mitochondrial homeostasis via the AMPK–MFF/Drp1 axis. When Drp1-mediated fission/mtROS/metabolic shifts reduce barrier integrity and mucosal oxygen consumption, the consequent changes in luminal oxygen tension and substrate niche drive microbiota compositional and metabolic shifts, which in turn feed back to alter metabolite output, forming a potential amplifying loop [87,88].

**Figure 3:**
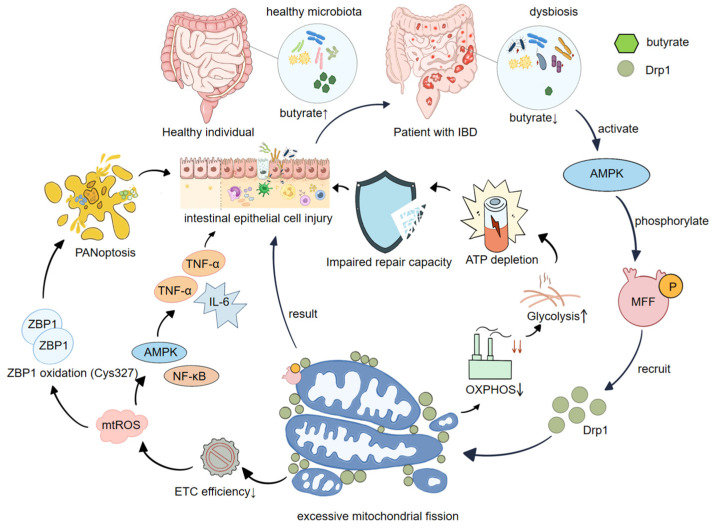
In healthy individuals, a balanced microbiota produces sufficient butyrate to maintain normal mitochondrial dynamics. In IBD patients, dysbiosis reduces butyrate, leading to energy stress and AMPK activation. AMPK phosphorylates MFF, promoting Drp1 recruitment and excessive mitochondrial fission. This results in two parallel branches: (1) mtROS accumulation activates NF-κB and ZBP1-dependent PANoptosis, increasing pro-inflammatory cytokines (TNF-α, IL-6); (2) OXPHOS impairment with compensatory glycolysis upregulation causes ATP depletion and reduced repair capacity. Both branches converge on intestinal epithelial barrier injury (tight junction disruption, increased permeability). Barrier damage further elevates mucosal oxygen tension, favoring facultative anaerobe expansion and worsening butyrate deficiency, thus forming a positive feedback loop. ↑ indicates increased production or elevated efficiency, ↓ indicates decreased production or reduced efficiency, and ↓↓ indicates severely decreased production or severely reduced efficiency.

We have prepared Figure 3 to provide a summary illustration of how Drp1-mediated mitochondrial fission influences gut microbiota homeostasis in IBD.

## 6. Drp1-Associated Mitochondrial Dynamics Imbalance: Perspectives on Signal Integration and Intervention

### 6.1. Alterations of Mitochondrial Fission–Fusion Dynamics in IBD and Inflammatory Diseases

Mitochondrial fusion and fission are key processes in mitochondrial dynamics. Their dynamic balance is crucial for maintaining cellular and organismal homeostasis, and disruption of this balance has been observed in various diseases. Mfn1 and Mfn2 mediate outer membrane fusion, while Opa1 mediates inner membrane fusion and participates in the maintenance of cristae structure. Mitochondrial fusion contributes to the repair of damaged mitochondrial DNA (mtDNA) and enhances energy metabolic efficiency [91]. The Drp1 protein primarily mediates mitochondrial fission. Cytosolic Drp1 is recruited to the mitochondrial surface by outer membrane receptors including Mff, MiD49, MiD51, and Fis1, where it forms a helical structure that constricts the mitochondrion, generating smaller mitochondrial units. Aberrant expression or function of these key proteins is associated with the pathogenesis of multiple diseases. Under physiological homeostasis, fusion facilitates the mixing of mitochondrial contents, allowing functional mitochondria to restore defective mitochondria through the sharing of RNA or proteins. On the other hand, fission increases mitochondrial number to supply sufficient mitochondria for cell division, while also enabling selective autophagic removal of damaged mitochondria to control mitochondrial quality within cells [92]. As summarized by Westermann and colleagues, fusion and fission determine mitochondrial morphology, and the balance between them dictates cellular state. Under inflammatory conditions, this balance often shifts toward fission, leading to mitochondrial network fragmentation, decreased oxidative phosphorylation efficiency, and increased production of ROS [93,94].

In IBD, multiple studies indicate that mitochondria may exhibit features of enhanced fission accompanied by limited fusion. As the core executor of mitochondrial fission, Drp1 can be regulated through multiple signaling pathways, including ubiquitination, SUMOylation, or phosphorylation, thereby affecting mitochondrial DNA stability and controlling mitochondrial quality, ultimately leading to apoptosis. However, it should be noted that mitochondrial fission is not a sufficient condition for apoptosis [93,95,96]. In studies of IBD and other chronic intestinal disorders, an increasing number of reports have documented abnormalities in mitochondrial morphology and function. For instance, several studies have observed that in the intestinal mucosa of patients with ulcerative colitis and Crohn’s disease, as well as in DSS-induced UC mouse models, mitochondria appear swollen, with disrupted cristae structure and decreased activity of respiratory chain complexes, accompanied by aberrant expression of fusion and fission proteins [8,96].

At present, studies directly focusing on Drp1 in IBD remain limited. However, considering the recurrent observation of imbalanced mitochondrial dynamics in both patients with IBD and animal models, Drp1-mediated enhancement of mitochondrial fission is likely an integral component of inflammation-associated mitochondrial remodeling rather than an isolated event. Importantly, it should not be simply interpreted that Drp1 acts as a singular driver of IBD pathogenesis. A more plausible explanation is that, within the inflammatory microenvironment of IBD, enhanced mitochondrial fission coupled with restricted fusion leads to disruption of mitochondrial dynamic homeostasis. Drp1 not only functions as the executor of fission but also may act as a hub linking inflammatory signaling and mitochondrial morphological remodeling. This may cooperatively promote further mitochondrial functional decline, metabolic reprogramming, and amplification of inflammatory responses, providing a structural basis for subsequent immune imbalance and barrier dysfunction.

### 6.2. Signaling Pathways Regulating Drp1 Activity: Convergence Nodes of Inflammatory and Metabolic Signals

#### 6.2.1. Regulation of Drp1 by Metabolic Sensing Pathways

AMPK Signaling Pathway: The AMP-activated protein kinase (AMPK) signaling pathway represents a classical energy-sensing mechanism that is activated upon ATP depletion or an increased AMP/ATP ratio, promoting catabolic processes while inhibiting anabolic metabolism. Under conditions of energy stress, in cell types such as U2OS cells, mouse embryonic fibroblasts (MEFs), and neurons, AMPK can directly phosphorylate mitochondrial fission factor (MFF), thereby recruiting Drp1 to the mitochondrial surface and initiating mitochondrial fission [76]. In contrast, another study reported that in the context of non-alcoholic fatty liver disease (NAFLD), AMPK activation, by upregulating p-Drp1 (Ser616) and downregulating p-Drp1 (Ser637), inhibited Drp1-mediated excessive mitochondrial fission, alleviating palmitic acid (PA)-induced hepatocyte injury and lipid accumulation [97].

Taken together, these findings indicate that AMPK-mediated regulation of Drp1 is context-dependent. Under severe stress, AMPK signaling can efficiently recruit Drp1 via MFF phosphorylation to fragment severely damaged mitochondria, preventing the disruption of the entire mitochondrial network. Whereas under chronic stress, when mitochondria are already in a pathological state of excessive fission, AMPK signaling can modulate Drp1 phosphorylation to suppress over-fission, thereby correcting pathological conditions, restoring mitochondrial function, and reducing oxidative stress and lipid accumulation. In IBD, the intestinal mucosa is persistently exposed to energy stress and metabolic dysregulation, suggesting that aberrant AMPK signaling may regulate Drp1 activity, although the precise directional effect remains to be further validated.

mTOR Axis: It is noteworthy that Drp1 is not the sole determinant of mitochondrial morphological remodeling, as mitochondrial fusion processes are equally critical. Verdejo et al. (2025) [98] reported in cardiomyocytes that mTOR inhibition can induce mitochondrial fragmentation through a Drp1-independent pathway. Specifically, mTOR inactivation promotes ubiquitination and degradation of prohibitin 1 (PHB1), activates OMA1, and induces OPA1 cleavage, thereby impairing mitochondrial fusion capacity [98]. This suggests that, in the context of inflammation and metabolic dysregulation, alterations in mitochondrial dynamics may involve both enhanced fission and restricted fusion. Therefore, when analyzing Drp1 regulatory networks, it is necessary to interpret its role within the broader framework of overall mitochondrial dynamic balance.

#### 6.2.2. Inflammation-Associated Kinases and Calcium Signaling Pathways

ERK1/2: Oncogenic Ras mutations, such as HRas^G12V, can activate the downstream Raf-MEK-ERK1/2 cascade, i.e., the MAPK pathway, and the activated ERK2 kinase directly phosphorylates Drp1 at the Ser616 residue, thereby inducing mitochondrial fission [21].

PKCδ Pathway: Under oxidative stress conditions, such as those caused by hypertension-induced brain injury, activated PKCδ forms a complex with Drp1 and translocates to mitochondria. It phosphorylates Drp1 at Ser579, inducing excessive mitochondrial fission and consequently leading to neuronal damage [99].

Ca^2+^–Calcineurin Pathway: Upon activation, the calcium-dependent phosphatase calcineurin dephosphorylates Drp1 at Ser637, resulting in Drp1 activation and subsequent mitochondrial over-fission [20,100].

**Figure 4:**
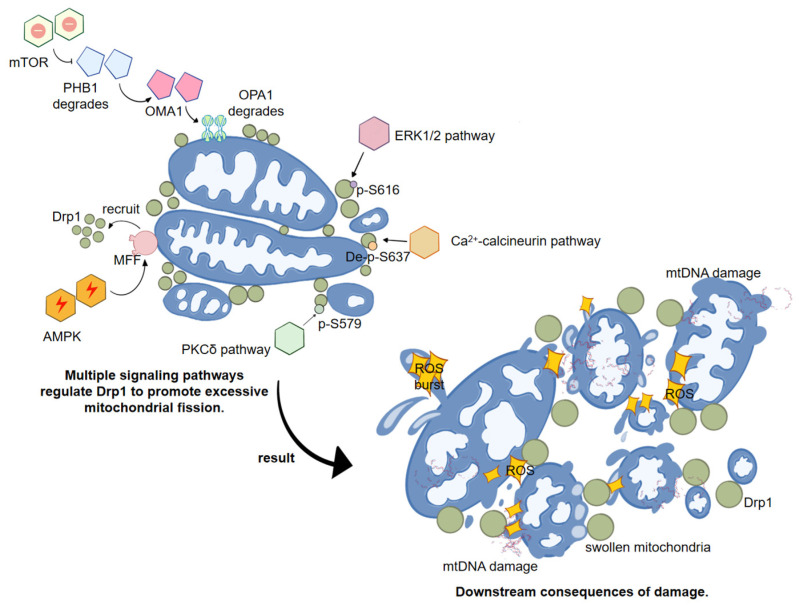
The illustration depicts the potential Drp1 signaling pathways and the downstream consequences of excessive mitochondrial damage.

We have prepared Figure 4 to provide a summary of the above content.

#### 6.2.3. Drp1 as a Signal Integration Node Linking Mitochondrial Dynamics Dysregulation to Disease

Collectively, these mechanisms indicate that metabolic sensing pathways, inflammation-associated kinases, and calcium signaling pathways can all regulate Drp1 expression or post-translational modifications through distinct mechanisms, thereby affecting mitochondrial function and morphological integrity. Accordingly, Drp1 serves as a central integrator of cellular metabolic perturbations and inflammatory signals, with its activity directly influencing the balance of mitochondrial dynamics. It should be emphasized that Drp1 functions as a signal integration node rather than as a master regulator. In the complex context of IBD, delineating the cell type-specific regulation of Drp1 by these signaling axes will be instrumental for a more precise understanding of the spatiotemporal features of mitochondrial dynamics dysregulation.

At the same time, the current evidence base remains heterogeneous and should be interpreted with caution. First, the mechanistic support for Drp1 in IBD is not equally strong across all cellular contexts. The most direct evidence is currently found in IECs, whereas the available data in macrophages are more suggestive of inflammatory amplification, and the evidence in adaptive immune cells remains comparatively indirect. Second, several mechanistic inferences in this field are derived from non-IBD systems or other inflammatory disease models, which may not fully recapitulate the spatial, metabolic, and immunological complexity of human IBD. Third, some upstream regulatory pathways, such as AMPK signaling, appear to influence Drp1 in a context-dependent manner rather than through a fixed unidirectional mechanism. Finally, interpretation of pharmacological studies requires particular caution, as commonly used compounds such as Mdivi-1 are not fully specific inhibitors of Drp1 and may exert Drp1-independent effects on mitochondrial respiration. Taken together, these limitations suggest that Drp1 is more appropriately viewed as a context-dependent signal-integrating node than as a universal or solitary driver of IBD pathogenesis.

### 6.3. Drp1-Targeted Interventions: Experimental Evidence and Translational Challenges

#### 6.3.1. Existing Animal and Cellular Models of Drp1 Knockout or Inhibition in IBD Research

In the study by Ye et al. (2024) [8], animal models of Drp1 conditional knockout were established, including intestinal epithelial cell (IEC)-specific Drp1 heterozygous knockout mice (Drp1-hetCKO, Villin-Cre^+^/Drp1^f^/^f+^) and macrophage-specific Drp1 knockout mice (LysM-Cre^+^/Drp1^f^/^f^). The IEC-specific model was generated by crossing Drp1 flox mice with Villin-Cre mice, whereas the macrophage-specific model was generated by crossing Drp1 flox mice with LysM-Cre mice. For cellular models, Drp1 expression in human intestinal epithelial cell lines (NCM460) was suppressed either by siRNA-mediated knockdown or pharmacologically using Mdivi-1. Similarly, Kang et al. (2025) [7] employed siRNA or pharmacological inhibitors (Mdivi-1 or P110) to inhibit Drp1 expression in human colorectal adenocarcinoma cells (HT-29). The authors noted that IEC-specific homozygous knockout mice could not be obtained, confirming the essential role of Drp1 in development [7,8].

#### 6.3.2. Drp1 Inhibitors

Mdivi-1 is one of the most commonly used small-molecule Drp1 inhibitors in recent years, directly targeting the GTPase activity of Drp1. In various cellular and animal models, Mdivi-1 has been reported to reduce mitochondrial fission, increase mitochondrial length, improve membrane potential, and decrease ROS production, suggesting its utility as an experimental tool for assessing Drp1-mediated fission [101,102]. It can cross the blood–brain barrier, modulate helper T cell activity, and suppress the development of experimental autoimmune encephalomyelitis [69]. However, Mdivi-1 exhibits limited efficacy in inhibiting the GTPase activity of purified Drp1, implying that its cellular effects may involve additional targets or indirect mechanisms [85].

P110 is currently one of the most widely studied peptide inhibitors targeting Drp1. Unlike Mdivi-1, it does not directly inhibit Drp1 GTPase activity but suppresses pathological mitochondrial fission by blocking the interaction between Drp1 and Fis1 [103]. Compared to Mdivi-1, P110 exhibits higher selectivity and does not interfere with the physiological functions of Drp1. In the context of IBD, P110 is considered a highly promising lead compound, with its high selectivity suggesting reduced side effects and providing a potential direction for the development of novel therapeutic strategies [7].

In recent years, other small-molecule Drp1 inhibitors, such as Drp1i27 and Drpitor analogs, have also been identified, which interact with the GTPase domain of Drp1 in a manner distinct from Mdivi-1 to inhibit its activity. Nevertheless, these molecules remain in the developmental and screening stages, with limited preclinical validation and sparse supporting literature [103].

Overall, current Drp1-targeting strategies are primarily employed for mechanistic investigations rather than as mature clinical candidates. In chronic inflammatory conditions such as IBD, a major challenge for future research remains the precise modulation of pathological mitochondrial fission while preserving baseline physiological Drp1 functions.

Additionally, we have created Table 3 to summarize the above content.

## 7. Translational Relevance and Clinical Implications

### 7.1. Why Drp1 Is Translationally Relevant in IBD

The clinical assessment of IBD has long faced a structural dilemma: clinical symptoms do not always synchronize with mucosal inflammation or histological activity, whereas endoscopy or biopsy cannot be used as a frequent monitoring tool. Therefore, international consensus strategies represented by the treat-to-target approach have proposed clinical remission and improvement in biochemical indicators (such as CRP and fecal calprotectin) as short- to mid-term targets, while endoscopic mucosal healing is regarded as a longer-term target aimed at reducing the risk of relapse and complications [104]. Recent evidence-based guidelines from the American Gastroenterological Association (AGA) further clarify that, during follow-up of patients with UC or CD, fecal calprotectin (FC) and serum C-reactive protein (CRP) can be used to monitor inflammatory activity and reduce unnecessary routine endoscopy. When biomarker results are inconsistent with the patient’s current clinical status, endoscopy should be considered to directly evaluate intestinal conditions [105]. At present, clinical practice has established a relatively stable monitoring framework: FC/CRP for routine surveillance, and endoscopy for confirmation in key situations [106]. Overall, the clinical value of FC and CRP is well established. FC correlates with neutrophil infiltration in the intestinal mucosa and can effectively reflect the local inflammatory burden of the intestine, thereby helping monitor the risk of relapse. CRP, in contrast, reflects systemic inflammation; however, its responsiveness varies among some patients with IBD—particularly those with UC—and reliance on CRP alone may lead to inaccurate estimation of mucosal inflammatory activity [107]. In this review, the goal of identifying better biomarkers is not to replace FC/CRP, but rather to complement their limitations. FC and CRP mainly answer the question of how intense the inflammation is, but they are less capable of explaining which key pathological execution mechanisms drive inflammation and why certain patients are more prone to persistent mucosal injury or recurrence. This is also why multi-biomarker panel strategies and mechanism-based stratification approaches have received increasing attention in recent years [108].

As discussed earlier in this review, mitochondrial homeostasis forms the biological foundation of intestinal epithelial barrier integrity and mucosal immune stability. When mitochondrial dynamics become imbalanced, energy metabolism is restricted, and oxidative stress increases, epithelial cells become more prone to enter cell-death programs and barrier collapse, thereby amplifying microbial-associated stimulation and inflammatory cascades [96,109]. Within this causal framework, Drp1-mediated mitochondrial fission represents one of the central regulatory nodes. Experimental and translational evidence from IBD/colitis studies has suggested that, under inflammatory conditions, the expression of fission-related molecules in colonic tissues (including Drp1 and Fis1) and/or the phosphorylation level of Drp1 increases. At the same time, mitochondrial network fragmentation can be observed in epithelial cells and macrophages. Inhibition of Drp1–Fis1-driven mitochondrial fission (for example with the peptide inhibitor P110) has been shown to alleviate the severity of DSS- or DNBS-induced colitis [8]. If abnormal mitochondrial fission in IECs is associated with complex programmed cell death (such as PANoptosis) and inflammatory amplification, and this relationship has been validated in both animal models and clinical samples, then the chain of “Drp1–fission–barrier injury–inflammatory amplification” may reveal a potential new therapeutic direction [8]. This perspective is consistent with previous reviews proposing that mitochondrial network dynamics participate in the pathogenesis of colitis and that inhibition of mitochondrial fission may represent a novel therapeutic strategy [110].

### 7.2. Therapeutic Targeting: Opportunities and Current Limitations

Within the current therapeutic landscape, treatment strategies generally favor suppressing immune amplification, with mucosal healing as the long-term target [104]. However, clinical challenges have not disappeared. Anti-TNF therapy still shows both primary non-response and secondary loss of response, requiring repeated adjustments of treatment strategies through therapeutic drug monitoring, dose escalation, switching within the same class, or switching to drugs with different mechanisms of action [111,112,113]. For UC, the JAK inhibitor tofacitinib was demonstrated in the phase III OCTAVE trials to be effective for both induction and maintenance of remission [114]. Multiple cohort studies have also shown its effectiveness with a manageable safety profile. Nevertheless, loss of response following dose reduction is not uncommon, indicating that in some patients the underlying pathological drivers cannot be fully “reset” solely by suppressing the inflammatory amplification arm [115]. In this context, extending the therapeutic perspective from immune amplification toward barrier injury better reflects the pathological characteristics of refractory and relapsing subtypes, and is also consistent with guideline recommendations emphasizing stratified and individualized treatment strategies [116].

The reason why Drp1-mediated mitochondrial fission is considered a potential therapeutic target does not lie in it being another anti-inflammatory target, but rather in the fact that it converges multiple pathogenic pathways at a common node. In the context of colitis, mitochondrial network fragmentation can be observed in epithelial and myeloid cells, accompanied by increased expression of fission-related molecules (such as Drp1 and Fis1) and enhanced Drp1 activation. This imbalance in mitochondrial dynamics is often accompanied by metabolic collapse, amplification of mtROS, cell death, and barrier disruption, thereby pushing inflammation from a reversible amplified state toward structural injury. Importantly, this pathway has already provided intervention-relevant and potentially translatable evidence. Blocking the pathological interaction between Drp1 and Fis1 can alleviate disease severity in DSS- and DNBS-induced colitis models, while preserving mitochondrial network integrity in epithelial cells and macrophages [43]. Recent studies have further linked intestinal epithelial cell (IEC) mitochondrial fission with complex programmed cell death, such as PANoptosis, and parallel validation in animal models and clinical samples suggests that targeting the Drp1 axis may represent a specific therapeutic entry point for interventions at the barrier execution layer [58]. Therefore, the Drp1 axis may be better positioned as a module complementary to existing immune-targeted therapies. When patients show improvement in inflammatory indicators but still experience delayed mucosal repair, frequent relapse, or refractory disease, inhibiting the “fragmentation–mtROS–cell death–barrier collapse” cascade may provide additional benefit rather than simply intensifying immunosuppression.

At the level of drug development and interpretation of evidence, two limitations must be clearly recognized in this review; otherwise, the Drp1 axis may be misinterpreted. First, mdivi-1 has long been widely used as a “Drp1 inhibitor”, yet mechanistic studies indicate that at commonly used experimental concentrations it significantly inhibits mitochondrial complex I-dependent oxygen consumption and ROS production, and its effects are not equivalent to Drp1 deficiency or genuine inhibition of mitochondrial fission. Therefore, the results involving mdivi-1 should be interpreted as reflecting intervention in mitochondrial respiration or oxidative stress, rather than being directly extrapolated as evidence of Drp1-specific targeting [85]. Second, Drp1 participates in physiological mitochondrial fission and mitochondrial quality control, and complete blockade of fission would impose costs on cellular homeostasis. Thus, a more translationally promising strategy is selective inhibition of stress-induced pathological fission while preserving physiological fission. The design of P110 reflects this concept: by interfering with the stress-dependent interaction between Drp1 and Fis1, it suppresses excessive mitochondrial fission [103]. Building on this foundation, structural analysis of the P110 binding interface has facilitated small-molecule drug discovery. Researchers have identified allosteric pockets on Drp1 suitable for modulation and screened small molecules capable of mimicking the beneficial effects of P110, thereby providing a more clinically relevant evidence chain for drug development aimed at inhibiting pathological fission without disrupting physiological fission [117].

When integrating the above evidence into clinical strategies, disease staging and stratification remain appropriate approaches. During the acute active phase, rapid suppression of inflammatory amplification should remain the primary goal, and advanced therapies may be applied when necessary, with loss of response addressed through therapeutic drug monitoring and management of immunogenicity [111,113,116]. In contrast, during the maintenance phase of remission, or in patient subtypes where inflammation has decreased but mucosal repair remains insufficient, the Drp1–mitochondrial fission axis may serve as a direction for physical barrier-oriented intervention. This framework may help explain and potentially interrupt the cycle of deterioration and relapse that occurs after initial inflammatory control, and provides the basis for the evaluation strategy described in Section 7.3.

### 7.3. Clinical Stratification and Future Validation Framework

Since the risk of delayed mucosal healing and frequent relapse may still persist even after inflammation has been suppressed or approaches remission, a validated strategy can be used to address this problem—namely the framework of the non-invasive disease severity index (non-invasive DSI). In this approach, the endoscopic component of the DSI is replaced with fecal calprotectin (fCal) or fecal myeloperoxidase (fMPO), and prospective cohort studies have directly demonstrated its ability to predict long-term complex disease courses. Specifically, baseline DSI-fCal and DSI-fMPO predicted a 24-month complicated disease course with AUROC values of 0.83 and 0.80, respectively. Moreover, in multivariable models, DSI-fCal ≥ 28 was independently associated with complicated disease course (aOR = 6.04, 95% CI 2.42–15.08), and DSI-fMPO ≥ 25 was also independently associated (aOR = 7.84, 95% CI 2.96–20.73) [108]. These findings provide an important conceptual framework: rather than debating which single biomarker is best, researchers should use quantifiable outcomes (such as complicated disease course, treatment escalation, hospitalization, or surgery) to evaluate whether a new measurement variable provides incremental predictive value.

Within this framework, we propose that Drp1 should not be described simply as another inflammatory biomarker, but rather be viewed as an indicator related to mucosal repair capacity, for which supporting evidence already exists. First, human and mechanistic studies show that excessive mitochondrial fission in colonic epithelial cells suppresses mucosal repair. One of the core mechanisms involves impaired epithelial butyrate metabolism and intestinal stem cell proliferation. In mouse models, inhibition of mitochondrial fission promotes mucosal healing, while enhanced mitochondrial fission has also been observed in the colonic epithelium of patients with UC [42]. This suggests that the Drp1–fission axis is directly associated with repair dynamics rather than simply reflecting the degree of inflammation. Second, Drp1-mediated excessive mitochondrial fission lies at the terminal stage of the “cell death–barrier collapse” cascade. Barrier disruption subsequently amplifies inflammatory stimulation, thereby forming a self-reinforcing pathogenic cycle. In studies combining colitis models with clinical samples, epithelial mitochondrial fission has been shown to drive ZBP1-dependent PANoptosis and is positively correlated with disease severity; targeting this pathway can alleviate colitis phenotypes [8]. Together, these two lines of evidence place the clinical relevance of Drp1 at a very specific point: it may better explain why mucosal repair remains delayed or relapse occurs despite biochemical improvement, rather than replacing FC or CRP as inflammatory markers.

Therefore, the most feasible strategy is to incorporate Drp1 as an additional measurement variable within existing key nodes, rather than proposing an entirely new screening workflow. When patients are already in clinical scenarios that require endoscopic or biopsy confirmation (for example, biochemical improvement accompanied by delayed healing or frequent relapse), epithelial Drp1-associated mitochondrial fission phenotypes could be assessed from the same biopsy samples. Following the validation paradigm used for non-invasive DSI, researchers could then evaluate whether this variable predicts disease progression and whether the Drp1 axis provides incremental predictive value beyond existing models such as DSI-fCal/DSI-fMPO or FC/CRP-based frameworks (for example, through ΔAUROC or independent associations with repair failure under comparable inflammatory burden). In this way, a reproducible and evidence-supported validation pathway could be established based on the existing results [8,42,108]. Overall, this section is intended to highlight the translational relevance and potential clinical implications of marine-derived bioactives, rather than to imply immediate clinical applicability.

## 8. Conclusions and Future Perspectives

Overall, current evidence suggests that Drp1-mediated mitochondrial fission is closely linked to epithelial barrier injury, inflammatory amplification, and intestinal microenvironmental remodeling in IBD. Even when immunosuppression is sufficiently strong, stable mucosal repair and a low risk of relapse are often difficult to achieve, and failure of anti-TNF therapy (including both primary non-response and secondary loss of response) occurs frequently [113]. When this structural dilemma is reconsidered at the mechanistic level, mitochondria appear more like the biological foundation of barrier maintenance and inflammatory thresholds, rather than merely a passive energetic background. Mitochondrial dysfunction intertwines with ROS accumulation, metabolic reprogramming, cell death, and increased epithelial permeability, ultimately determining whether epithelial injury can still remain within a reversible range [96,109].

However, the key problem is that “mitochondrial dysfunction” is itself an overly broad concept. The processes that truly translate diverse upstream stresses into observable and intervenable structural consequences are often the execution nodes of mitochondrial dynamics and quality control. For this reason, our focus centers on Drp1. As the core GTPase regulating mitochondrial fission, Drp1 determines whether stress inputs are amplified into mitochondrial network fragmentation and mismatched quality control. Its structural domains, cooperation with receptor proteins, and regulatory logic have been relatively well characterized at the fundamental level [118]. More importantly, recent IBD studies are moving Drp1 from a position of mere accompanying change toward a more central role. When excessive Drp1 activation triggers mitochondrial fragmentation together with impaired quality control, epithelial cells more readily cross the injury threshold and enter a barrier-collapse pathway driven by mtROS amplification, mtDAMP release, and concurrent activation of multiple cell-death programs. Taking UC as an example discussed earlier, “fission-mediated PANoptosis” has been proposed as a direct execution mechanism, and causal evidence has begun to emerge—such as the findings that saquinavir alleviates colitis by inhibiting Drp1 [8]. At the same time, studies demonstrating that the TFAM–mtDNA complex activates the myeloid STING–IL-12 axis further translate mtDAMPs from a theoretical danger signal into a pathway that can be detected and experimentally blocked [119]. Therefore, a more appropriate description is not to treat Drp1 as a new inflammatory biomarker, but rather to define it as an execution-level pressure node of structural injury and repair failure. It integrates multiple upstream stresses—including inflammation, metabolic perturbation, hypoxia, and microbial imbalance—into mitochondrial morphological and functional collapse. Through signaling networks such as mtROS–NLRP3, mtDNA–STING, and PANoptosis, these processes convert the residual risk remaining after inflammatory control into persistent barrier instability and recurrent disease activity [8,96,119].

Following this line of reasoning, what will ultimately determine whether Drp1 can move from theoretical explanation toward translational application is not simply demonstrating again that it “increases” or “participates”, but rather placing Drp1 within an evidence framework centered on temporal dynamics and activation thresholds. Mitochondrial fission itself is a double-edged process: moderate fission during repair and renewal may facilitate mitochondrial turnover and quality control. What truly drives epithelial cells toward irreversible collapse is the sustained high-level activation of Drp1 under combined inflammatory, hypoxic, and metabolic stress, together with a mismatch between mitochondrial fission and mitophagy/biogenesis, which then triggers a vicious cycle involving mtDAMP–STING/NLRP3 signaling and concurrent cell-death pathways [96,119]. Consequently, the most important questions for future research are: at what stage Drp1 activation occurs, in which epithelial cell populations or mucosal regions it appears, and at what level of activation tissues remain within a “repairable” injury state; and once this threshold is crossed, when the process shifts into an “irreversible” barrier-collapse trajectory. Such questions can only be reliably addressed through time-resolved endpoint chains within a unified experimental system: starting from morphological evidence of mitochondrial fission and fragmentation, sequentially linking downstream events including mismatched quality control, emergence of mtROS and mtDAMPs, concurrent activation of STING/NLRP3 signaling and PANoptosis, and ultimately returning to barrier function readouts and tissue repair outcomes, thereby completing a closed validation loop from mechanism to phenotype and finally to potential intervention [8,119].

At the level of clinical translation, consistent with the arguments above, we also propose avoiding the simplification of Drp1 as a single biomarker. Instead, it should be considered part of a composite evaluation framework for repair-failure risk, together with measurements of inflammatory burden and barrier injury. On one hand, such an approach may help identify patient groups that remain at high risk even after strong immunosuppression; on the other hand, its value should be validated through real clinical outcomes. Events such as relapse, treatment escalation, hospitalization, or surgery reflect structural repair more accurately than short-term symptom changes and are more closely aligned with the clinical reality of treatment failure in IBD [113]. Ultimately, if the temporal–threshold pattern of Drp1 activation can be consistently aligned with measurable pathways (such as TFAM–mtDNA–STING signaling and fission-associated PANoptosis) and its independent contribution can be validated within outcome-oriented stratification strategies, then Drp1 may move beyond mechanistic interpretation and become a translational node for patient stratification and combined therapeutic decision-making—but only when its temporal and threshold dynamics can be reliably measured and shown to predict relapse and repair failure.

## Figures and Tables

**Table 3:** Drp1 inhibition models and inhibitors.

Drp1 Inhibition Models and Pharmacological Inhibitors	Type	Advantage	Disadvantage	References
Drp1-hetCKO (Villin-Cre^+^/Drp1^f^/^f+^)	Intestinal epithelial cell (IEC)-specific Drp1 knockout mouse model	Intestinal epithelial cell-specific	-	[8]
LysM-Cre^+^ Drp1^f^/^f^	Macrophage-specific Drp1 knockout mouse model	Macrophage-specific	-	[8]
Mdivi-1	Quinazolinone compounds	Widely applicable and capable of crossing the blood–brain barrier	Difficult to inhibit the GTPase activity of purified Drp1	[101,102]
P110	Enzyme	Higher selectivity and does not interfere with the physiological functions of Drp1	Unable to inhibit the GTPase activity of Drp1	[7]
Drp1i27/Drpitor	Small-molecule inhibitors	Primarily used for mechanistic validation	Currently limited in application, used only for development and screening	[103]

## Data Availability

No new data were created or analyzed in this study. Data sharing is not applicable to this article.
